# SARS-CoV-2 Vaccination-Induced Transverse Myelitis

**DOI:** 10.7759/cureus.16624

**Published:** 2021-07-25

**Authors:** Nayha Tahir, Gowthami Koorapati, Sonika Prasad, Hafiz Muhammad Jeelani, Robin Sherchan, Jishna Shrestha, Maryna Shayuk

**Affiliations:** 1 Internal Medicine, Chicago Medical School, Internal Medicine Residency Program, Northwestern Medicine McHenry Hospital, McHenry, USA

**Keywords:** longitudinally extensive transverse myelitis, covid 19, sars-cov-2, bells palsy, urinary retention, covid 19 vaccine

## Abstract

While mass immunization against coronavirus disease 2019 (COVID-19) rolls out around the globe, safety concerns and adverse events that need prompt evaluation are also emerging. We report a case of transverse myelitis and Bell's palsy after receiving Johnson and Johnson COVID-19 vaccination under the emergency use authorization in a healthy young woman with no past medical history. Other possible etiologies of her symptoms were ruled out, and she was treated successfully with steroids and plasma exchange.

## Introduction

Coronavirus disease 2019 (COVID-19) caused by severe acute respiratory syndrome coronavirus 2 (SARS-CoV-2) was proclaimed as a pandemic on March 11, 2020 by the World Health Organization (WHO). By the end of June 2021, about 178 million confirmed cases and 3.8 million deaths have been reported worldwide according to WHO. To combat the pandemic, vaccines for COVID-19 started developing at an exceptional rate. Many of them were approved for emergency use by different regulatory authorities like the Food and Drug Administration in the United States, the Medicines and Healthcare Products Regulatory Agency in the United Kingdom, and the European Medicines Agency after reviewing clinical efficacy data from phase 3 results, which subsequently led to mass vaccination worldwide. While it is an excellent achievement amid the pandemic, the vaccines still have to undergo post-marketing surveillance to monitor common and rare adverse events that need to be reported. To our knowledge, there is no reported case of transverse myelitis (TM) and Bell's palsy secondary to the Johnson and Johnson COVID-19 vaccine. Thus, herein, we report a case of TM and Bell's palsy seen in a healthy young woman after receiving the Johnson and Johnson COVID-19 vaccine. 

## Case presentation

A 44-year-old previously healthy woman presented to the emergency department with a three-day history of worsening back pain associated with nausea and urinary retention. She also reported numbness and weakness in the lower extremities for the last two days along with low-grade fever, chills, and body aches for two to three days. Patient received the Johnson and Johnson COVID-19 immunization 10 days before presentation. Her family history was unremarkable for muscular disorders, multiple sclerosis, stroke, and any rheumatological disorders. 

On physical examination, blood pressure was 135/83 mmHg, heart rate of 78 beats per minute, temperature of 99.1 Fahrenheit, respiratory rate of 11 breaths per minute, and oxygen saturation of 99% on room air. She was awake, alert, attentive, and oriented to time and person and did not have any nuchal rigidity, meningismus, or Lhermitte's sign. Her cranial nerves II-XII examination was grossly intact. She had normal muscle tone and strength in both upper and lower extremities with normal coordination and gait. Exaggerated (3+) deep tendon reflexes were noted in bilateral upper and lower extremities and positive Babinski signs were present bilaterally. Sensory examination was abnormal with decreased vibration in bilateral toes, and mild paresthesia in the neck and abdomen. The rest of the physical examination was unremarkable. 

**Figure 1:**
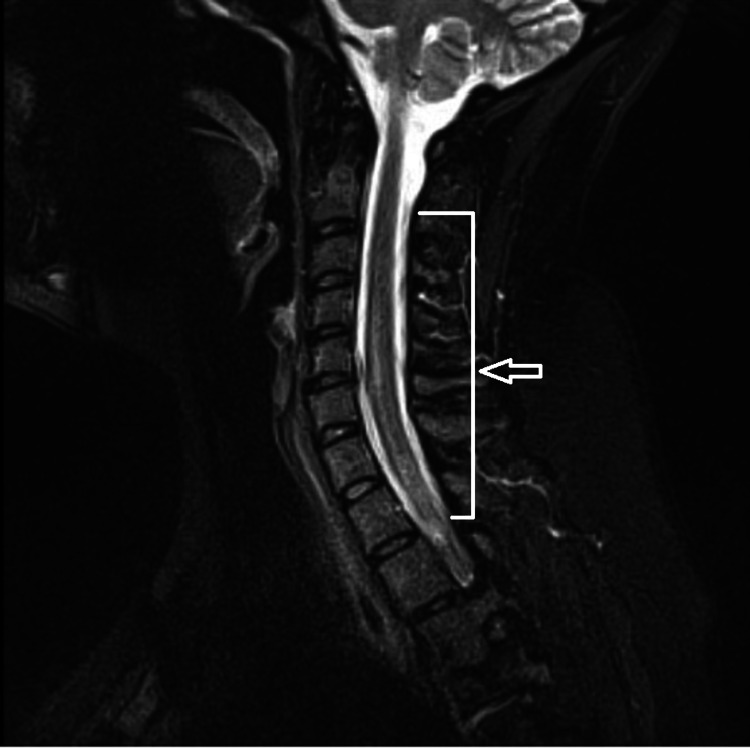
Magnetic resonance imaging of the cervical and upper thoracic spine with and without contrast showing a long segment of increased T2 signal within the central spinal cord extending at least from the C2-3 segment into the visualized thoracic spine (arrow).

Initial laboratory analysis showed an unremarkable complete metabolic profile, complete blood count, and a negative SARS-CoV-2 nucleic acid amplification test. The patient underwent magnetic resonance imaging (MRI) of the cervical, thoracic spine, and lumbar spine with and without contrast, showing increased signal throughout the spinal cord extending from the C2-3 segment into the upper thoracic spine (Figure 1).

 

Given significant findings in the cervical and upper thoracic spine, the patient underwent MRI brain with and without contrast, which was unremarkable for any inflammatory process. Neurology services evaluated her and recommended starting high-dose methylprednisolone therapy for the next three days. The patient required multiple straight catheterizations due to urinary retention. The next day she had significant improvement in her symptoms.

**Figure 2:**
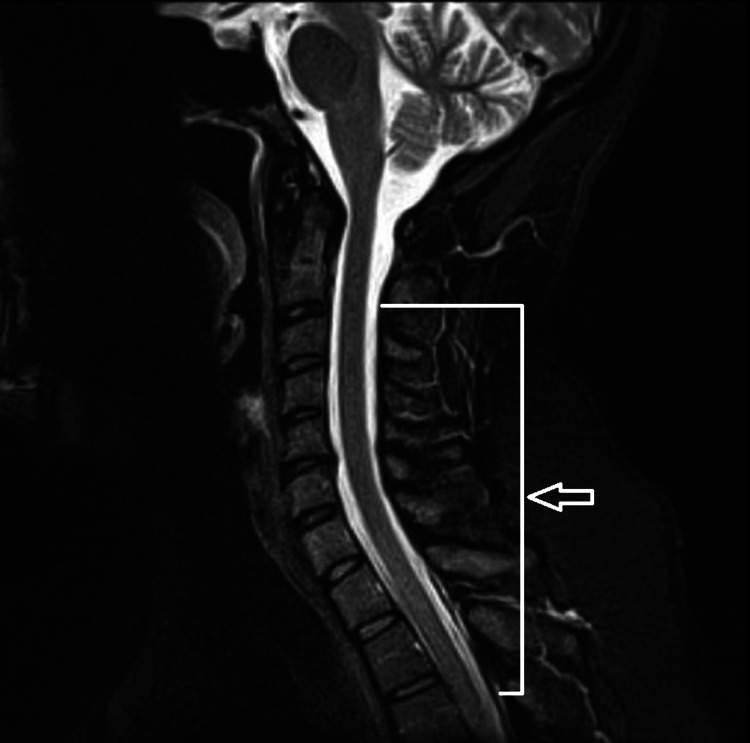
Magnetic resonance imaging of the cervical and upper thoracic spine with and without contrast showing a decreased T2 signal intensity of the cervical and upper thoracic region spinal cord, due to improving transverse myelitis after high-dose methylprednisolone therapy and plasmapheresis (arrow).

Lumbar puncture was performed with cerebrospinal fluid (CSF) analysis showing a white blood cell count of 227 µ/L, red blood cells of 25 µ/L, total cell count of 100 with 96% of lymphocytes, 3% of monocytes, and 1% of eosinophils. CSF chemistry analysis showed glucose of 71 mg/dL, protein of 43 mg/dL, albumin of 0.6 g/dL, and lactase dehydrogenase of 8 units/L. The rest of the workup, including the venereal disease research laboratory test, *Toxoplasma gondii* antibody, neuromyelitis optica IgG antibody, antinuclear antibody screen, and cryptococcal antigen, were negative. CSF bacterial, viral, and fungal cultures were sent and were later reported as negative. The myelin basic protein was noted to be 2.8 mcg/L, and the IgG index was 0.67. No oligoclonal bands were present. The CSF pathology showed small lymphocytes and rare monocytes. To prevent recurrence or exacerbation of myelopathy, neurology services decided to start her on plasma exchange for five treatments over 10 days after the completion of three days of pulse dose of methylprednisolone. While undergoing plasma exchange, she developed Bell's palsy, suggesting the persistence of the underlying inflammatory process despite three days of methylprednisolone and four sessions of plasma exchange. Repeat MRI cervical and thoracic spine with and without contrast was performed after five sessions of plasma exchange and showed a decrease in signal intensities in the cervical and thoracic spine depicting improvement in her condition (Figure 2).

The patient's symptoms of numbness, urinary retention, and Bell’s palsy resolved over 10 days of treatment with a pulse dose of steroids and five sessions of plasma exchange. The patient was discharged home with a recommendation to follow up neurologist and neuro-immunologist as an outpatient.

## Discussion

TM is a rare, acquired focal inflammatory disorder of the spinal cord in the absence of a compressive lesion. The most prevalent causes are demyelinating illnesses such as multiple sclerosis, neuromyelitis optica, infections, and vaccines. Systemic autoimmune diseases such as systemic lupus erythematosus and Sjögren's syndrome can damage the spinal cord in rare cases [1]. The cause of TM is unknown in up to 30% of instances, making it essential to distinguish idiopathic from disease-associated TM [2]. TM is clinically defined by the onset of acute or sub-acute motor, sensory, and autonomic dysfunction [3]. A recent literature review demonstrated cervical T2 signal anomaly in 44%, thoracic T2 signal abnormalities in 37%, and multifocal lesions in 5% of patients [4]. In our case, the MRI study demonstrated a long segment of increased signal throughout the spinal cord extending at least from C2-3 up to the thoracic spine, suggestive of TM. The role played by the SARS-CoV-2 vaccine, in this case, was found to be significant after ruling out other causes such as connective tissue disorders, vasculitis, infectious etiology, and multiple sclerosis. Acute TM affects between 1 to 8 million people per year, with a peak occurrence between the second and fourth decade of life [1,5]. Based on a systematic review of PubMed, EMBASE, and DynaMed journals published between 1970 and 2009, a total of 37 cases of vaccine-associated TM is 3.70, which is within the range predicted in the general population of the United States [6,7]. 

The idea of autoimmunity, where antibodies and T cells respond cross-reactively to central and peripheral nervous system neural epitopes, is emphasized in the hypothesis for vaccine-induced neuroinflammatory disease [8]. The "Molecular Mimicry" concept emphasizes that vaccination might cause autoimmune disease by microbial pathogen proteins similar to human proteins [8]. Only with the oral poliovirus vaccination, a pathogenic causal link for TM was identified [9]. A common denominator among vaccines such as an adjuvant may play a role in the pathogenesis of TM. According to Vaccine Adverse Event Reporting System (VAERS), 254 (2.69%) of the 9442 adverse events following immunization recorded in association with Pfizer-BioNTech, Moderna, and Johnson & Johnson's COVID-19 vaccines were neurological, with nine cases of TM reported in VAERS [10]. Furthermore, two ATM serious adverse events were reported with the ChAdOx1 nCoV-19 (recombinant) vaccine trials [11,12]. The SARS-CoV-2 structural surface vector glycoprotein antigen (spike protein; nCoV-19) gene is included in a replication-deficient chimpanzee adenoviral ChAdOx1 vaccine (AZD1222). The antigen may also be present in the COVID-19 vaccination AZD1222, or its chimpanzee adenovirus adjuvant could be a possible trigger leading to ATM [13]. Johnson & Johnson's COVID-19 vaccine incorporates the adenovirus, a prevalent cause of respiratory illnesses. The adenovirus's DNA is altered to form a critical component of the SARS-CoV-2 virus particle, to which the body responds with an immunological response [14]. This could be a possible immunological trigger for ATM. To our knowledge, this is the first reported longitudinally extensive TM following the SARS-CoV-2 vaccine with the lesion of TM extending for more than three vertebral segments in length [15]. Currently, there are no standard guidelines to treat TM secondary to the COVID-19 vaccine. Our patient was treated with intravenous steroids and plasma exchange and showed significant improvement in her symptoms [16,17]. 

## Conclusions

The COVID-19 vaccines were approved for emergency use based on phase 3 clinical efficacy data and they have to go through post-marketing surveillance. As mass immunization continues across the world, adverse events are expected to be increasingly reported. Numerous COVID-19 vaccine-related adverse events involving the nervous system were described in the available literature; however, TM and Bell's palsy have not been specifically reported. The physicians should be aware of this adverse effect after Johnson and Johnson's COVID-19 vaccination and maintain a high index of suspicion in patients coming with typical symptoms of TM after receiving the vaccine, report it, and treat it immediately.
